# Finite element analysis of the biomechanical effects of manipulation of lower limb hyperextension on the sacroiliac joint

**DOI:** 10.3389/fbioe.2025.1533585

**Published:** 2025-03-27

**Authors:** Bangmin Luo, Yikai Li, Cheng Wang, Zhun Xu

**Affiliations:** ^1^ Department of Spine Surgery, The First Affiliated Hospital, Hengyang Medical School, University of South China, Hengyang, Hunan, China; ^2^ School of Traditional Chinese Medicine, Southern Medical University, Guangzhou, Guangdong, China

**Keywords:** manipulation, biomechanical, sacroiliac joint, finite element analysis, stress, strain

## Abstract

**Objective:**

The objective of this study was to explore the effects of four Manipulations of lower limb hyperextension (MLLHs) on the sacroiliac joint (SIJ) and surrounding ligaments.

**Methods:**

A three-dimensional finite element model of the pelvis was built. Four MLLHs were simulated. The stresses on the pelvis and SIJ were calculated. The SIJ displacements and ligament strains were analyzed.

**Results:**

Under MLLH-F1, -F2, -F3 and -F4, the maximum stresses on the pelvis were 49.2, 50.5, 48.6 and 54.0 MPa, and the maximum stresses on the left SIJ were 3.1, 3.2, 3.0 and 3.4 MPa, respectively. The total SIJ displacements were 0.129, 0.164, 0.080 and 0.154 mm under MLLH-F1, -F2, -F3 and -F4, respectively. The four MLLHs all caused different degrees of ligament strain, MLLH-F2 the greatest.

**Conclusion:**

MLLH-F2 and -F4 caused greater stresses on the pelvis and the SIJ surface. The four MLLHs all produced small SIJ displacements. MLLH-F2 produced the largest SIJ displacement and the greatest ligament strain. These findings can guide the choice of therapy.

## 1 Introduction

The sacroiliac joint (SIJ) is the largest axial joint in human body. It connects the spine to the pelvis and transfers weight from the upper body to the lower extremities (Cohen, 2005; Joukar et al., 2018). The articular surface is composed of the ligament part and the synovial part. The ligament part maintains the stability of the SIJ, and the synovial part provides a certain range of motion of the SIJ (Al-Subahi et al., 2017; Poilliot et al., 2019). One side of the SIJ bears more weight because of the abnormal gait and is likely to degenerate (DonTigny, 1990; Vanelderen et al., 2010). Long-term incorrect standing or sitting posture can cause strain on the SIJ joints and surrounding ligaments, which can lead to lower back pain (Sacroiliac joint dysfunction: pathophysiology, diagnosis, and treatment, 2021). Recent studies have found that SIJ diseases can also cause low back pain, accounting for approximately 14.5%–22.5% of cases (Lindsey et al., 2014).

Commonly, abnormal gait and long-term strain cause high pressure in the SIJ and surrounding ligaments, resulting in the SIJ and ligament damage. Inflammation damages the SIJ. The range of motion of the SIJ increases abnormally after lumbar fusion surgery with fixation of the sacrum, which aggravates the strain of the joint. These factors may cause SIJ pain without specific causes (Schuit et al., 1989). The mechanism may include the following processes: these pathogenic factors acting on the auricular surface of the sacrum and ilium may cause injury to the ligaments or muscles around the SIJ, which will result in slight movement of the SIJ, making the joints difficult to reduce. The mechanical environment of the joints may ultimately be imbalanced, and the soft tissues will be damaged. This condition is one of the causes of SIJ dysfunction (Hing et al., 2015).

Clinically, SIJ dysfunction without specific cause is usually treated by manipulation (Farazdaghi et al., 2018; Kamali et al., 2019; Nejati et al., 2019; García-Peñalver et al., 2020). Many studies have reported that the manipulation of lower limb hyperextension (MLLH) could significantly relieve lower back pain in patients with SIJ dysfunction, and the treatment efficacy rate was 90%–95% (Xie, 2017; Liu et al., 2020; Fan and Wu, 2021). Manipulations have the characteristics of no trauma and a quick effect, so they are widely accepted by patients. In a previous study, a finite element pelvic model was built, and it was found that MLLH could produce small SIJ displacement and different degrees of ligament strain (Xu et al., 2020). However, the point and direction of the manipulative force were different among therapists. It is not known whether the MLLH with different points and directions will produce different effects on the SIJ. Thus, the purpose of this study is to explore the biomechanical characteristics of four MLLHs through a three-dimensional finite element model, so as to provide a reference for clinical manipulation.

## 2 Materials and methods

### 2.1 Model construction

A healthy male volunteer (34 years old, 170 cm in height, and 65 kg in weight) was recruited. The volunteer signed the informed consent form, and the study protocol was approved by the ethics committee. Computed tomography (CT) of the pelvis and femur with axial slices 0.5 mm thick was performed. CT data were imported into Mimics 20.0 (Materialise Company, Leuven, Belgium), and the cortical and cancellous regions of the bones were identified. The surfaces of the model were meshed using Geomagic 2013 (Raindrop Company, Marble Hill, USA). The SIJ consists of articular cartilage, the endplate of the sacrum and the ilium, and the surrounding ligaments. The sacral and iliac cartilage of the SIJ were built with uniform thicknesses of 2 mm and 1 mm, respectively. The ranges of the articular surfaces were derived from CT data. The thicknesses of the sacral and the ilial endplates were set at 0.23 mm and 0.36 mm, respectively. The gap width between the two cartilages was assumed to be 0.3 mm. (McLauchlan and Gardner, 2002; Kim et al., 2014). The hip joints were set to be fully constrained. The material properties, based on previous studies (Kim et al., 2014; Lee et al., 2017), are listed in Table 1.

**TABLE 1:** Material properties of the sacrum, ilium, femur, pubic symphysis and endplate.

Material		Young’s modulus (MPa)	Poisson’s ratio
Sacrum	Cortical	12,000	0.3
	Cancellous	100	0.2
Ilium	Cortical	12,000	0.3
	Cancellous	100	0.2
Femur	Cortical	15,000	0.3
	Cancellous	100	0.2
Pubic symphysis		5	0.45
Articular cartilage		100	0.3
Endplate		1,000	0.4

The anterior sacroiliac ligament (ASL), short posterior sacroiliac ligament (SPSL), long posterior sacroiliac ligament (LPSL), sacrospinous ligament (SS), interosseous sacroiliac ligament (ISL), and sacrotuberous ligament (ST) complexes were established as 3D tension-only truss elements. The attachment ranges were based on previous literature (Kim et al., 2014). The ASL is made up of numerous thin bands that span the ventral surface of the SIJ, connecting the lateral aspect of the sacrum to the margin of the auricular surface of the ilium. The LPSL extends from the posterior superior iliac spine to the third and fourth transverse tubercles of the back of the sacrum. The SPSL lies deep relative to the LPSL and consists of large fibers attaching the lateral aspect of the dorsal sacral surface to the tuberosity of the ilium. The ISL lies in the intra-articular space and is composed of a series of short, strong fibers connecting the tuberosities of the sacrum and ilium. The SS is a thin triangular ligament that connects the ischial spine to the lateral border of the sacrum. The ST is behind the sacrospinous ligament, which attaches the ischial tuberosity to the lateral border of the sacrum. The material properties of each ligament were obtained from the literature (Lee et al., 2017). In the end, the pelvis-femur model included 727,474 elements and 275,399 nodes.

### 2.2 Simulation of MLLHs

**FIGURE 1:**
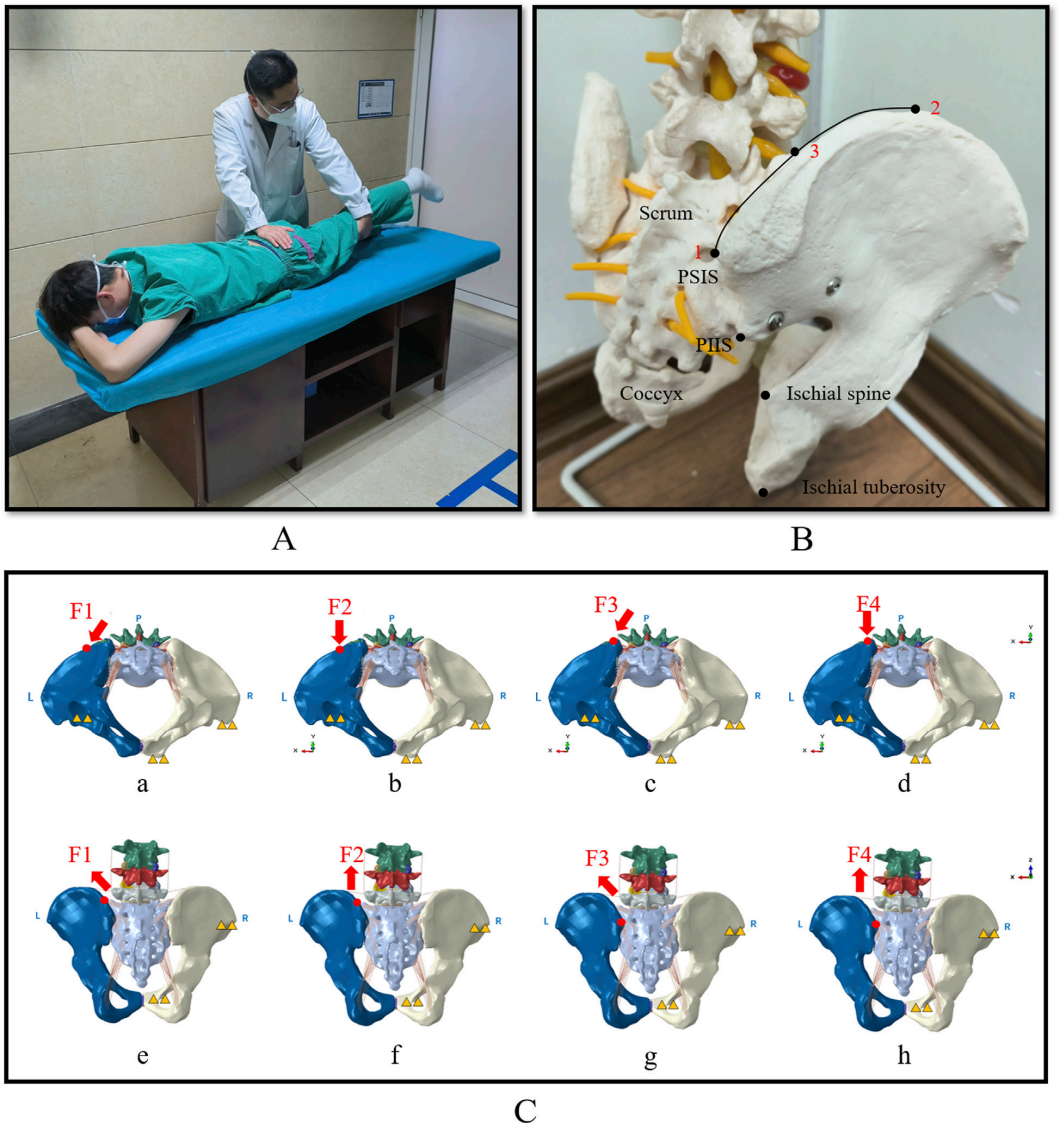
The picture showing MLLH, pelvic force application point and biomechanical modelling of the four loading conditions. **(A)** Showed that the therapist performed MLLH on the patient. **(B)** Showed the two force points in a pelvic model. Point 1 was the PSIS, which was the force point of MLLH-F3 and -F4. Point 2 was the highest point of the iliac crest. Point 3 was the mid-point between the highest point of the iliac crest and the PSIS, which was the force point of MLLH-F1 and -F2. **(C)** Showed the loading and boundary conditions for four MLLHs. The yellow triangles represent the fixed sites of pelvic model. The inferior view **(a–d)** and posterior view **(e–h)** of pelvis are shown. **(a, e)** MLLH-F1; **(b, f)** MLLH-F2; **(c, g)** MLLH-F3; **(d, h)** MLLH-F4. MLLH, manipulation of lower limb hyperextension; PSIS, posterior superior iliac spine; PIIS: posterior inferior iliac spine.

The patient lay in a prone position, and the leg treated was hyperextended at the hip so that the anterior superior spine could just lift off the bed. Then, the therapist applied a downward force to the treated iliac crest. The procedure is shown in Figure 1A.

The MLLH simulation was as follows: The magnitudes of the forces were determined by determining the manipulative power of five therapists using a biomechanical testing machine. Their average manipulative force was 600 N (Xu et al., 2020). In this manner, the right lateral region of the ilium and the right pubic tubercle were fixed. Then, a pushing force of 600 N along the dorsal-ventral direction was applied at the left iliac crest or posterior superior iliac spine (PSIS). PSIS is a bony prominence on the posterior aspect of the ilium, which facilitates the application of manipulative force by physiotherapists. The iliac crest is closer to the synovial portion of the SIJ, making it more likely to induce SIJ micromotion (Zhang et al., 2019; Xiao et al., 2022). Therefore, the PSIS and the midpoint between the highest point of the ilium and the PSIS were selected as the points of manipulative force. The articular surface of the SIJ forms an angle of approximately 30° with the sagittal plane of the human body (Zhang et al., 2019; Xiao et al., 2022). Based on the structure, two orientations were selected: one at a 30-degree angle relative to the sagittal plane to align with articular surface, and the other parallel to the sagittal plane. Additionally, the two points and orientations of manipulative force were determined based on the consensus of senior physiotherapists (Zhong et al., 2025; Huang et al., 2019; Fan and Wu, 2021). The two force points are described in Figure 1B.

Four MLLHs were tested. MLLH-F1: The force was applied at the left iliac crest (Point 3, Figure 1B) at an angle of 30° from the sagittal plane, which was roughly parallel to the SIJ surface. MLLH-F2: The force was applied at the left iliac crest (Point 3, Figure 1B), parallel to the sagittal plane. MLLH-F3: The force was applied at the left PSIS (Point 1, Figure 1B) at an angle of 30° from the sagittal plane. MLLH-F4: The force was applied at the left PSIS (Point 1, Figure 1B), parallel to the sagittal plane. The detailed loading and boundary conditions, as well as the x-, y-, and z-axes, are described in Figure 1C. The compressive stresses and displacements of the SIJ and the strains of the ligaments with the four MLLHs were then investigated using Abaqus 2018 (Dassault Systemes SA, Massachusetts, USA).

### 2.3 Mesh convergence study

To assess the accuracy of the pelvic model, a convergence analysis was performed through systematic mesh refinement. Four finite element models with progressively decreasing element sizes were constructed, with corresponding element and node counts detailed in Table 2. The models incorporated boundary conditions, material properties, loading configurations, and constraint definitions as previously described in the abovementioned section. Each mesh configuration was subjected to four loading conditions (MLLH-F1, -F2, -F3 and -F4). Subsequently, the maximum stresses and displacements observed on the left SIJ articular surface of the sacrum were comparatively analyzed across all model variants under the four loading scenarios.

**TABLE 2:** Element and node numbers for four different mesh resolutions.

Model	Element number	Node number
Mesh 1	204094	75352
Mesh 2	378199	133863
Mesh 3	727474	275399
Mesh 4	1590376	589032

### 2.4 Model validation

Two tests were performed to validate the model. For the pelvic model, the distribution of the principal strain of the pelvis was compared with that indicated by Zhang et al. (2010). Zhang et al. analyzed the distribution of principal strain on the cortical bone of the pelvis in a single-legged stance. In this model, the distribution of the principal strain of the pelvis was observed under the same loading and boundary conditions.

For the sacrum model, the relationship between load and displacement was compared with that reported in cadaveric (Miller et al., 1987) and computational studies (Eichenseer et al., 2011; Kim et al., 2014). In the cadaveric experiment, the bilateral ilia were fixed. Five translational forces (anterior, posterior, superior, inferior, and mediolateral) of 294 N and three moments (flexion, extension, and axial rotation) of 42 Nm were applied separately to the center of the sacrum. Under these stimuli, the displacements of a node lying in the mid-sagittal plane between the inferior S1 and superior S2 vertebral endplates were calculated. In this model, the displacement was estimated under the same loading.

## 3 Results

### 3.1 Mesh convergence study

**FIGURE 2:**
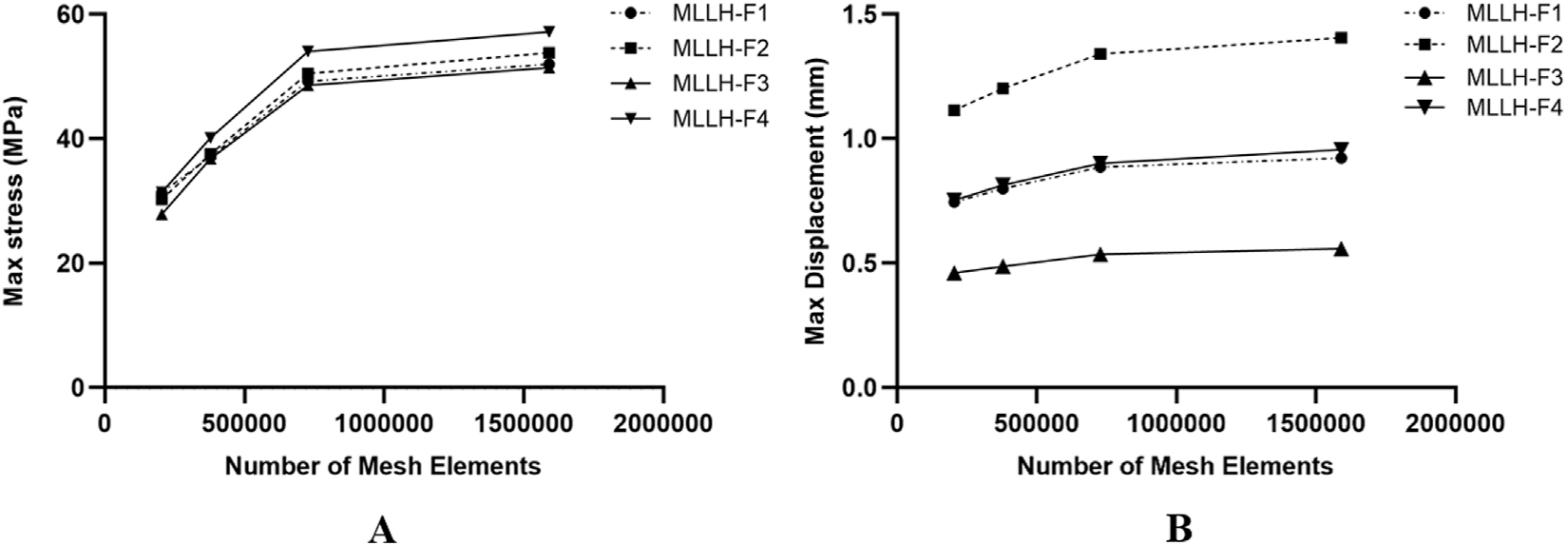
The picture of the maximum stresses and displacements of the left SIJ articular surface of the sacrum under four MLLHs. **(A, B)** Showed the maximum stresses and displacements on the left SIJ surface of the sacrum for different number of mesh elements, under MLLH-F1, -F2, -F3, and -F4, respectively. SIJ, sacroiliac joint; MLLH, manipulation of lower limb hyperextension.

Quantitative evaluation of maximum stresses and maximum displacements on the left SIJ surface of the sacrum was performed across all mesh configurations under four loading conditions (MLLH-F1, -F2, -F3 and -F4), which are shown in Figure 2. Comparative analysis revealed less than 5% variation in maximum stress and maximum displacement between Mesh 3 and Mesh 4 across all loading scenarios, falling within acceptable convergence thresholds (≤5%). Based on these findings, Mesh 3 (727,474 elements) demonstrating optimal balance between computational efficiency and solution accuracy was subsequently adopted for subsequent biomechanical analyses.

### 3.2 Model validation

**FIGURE 3:**
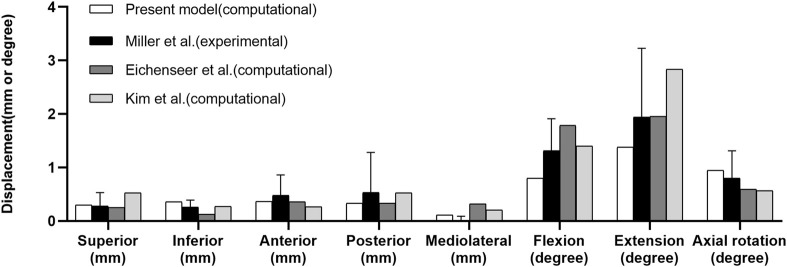
Comparison of sacral displacements under eight loadings comparable to those in previous experimental and computational studies.

The principal stresses were distributed mainly in the upper and posterior areas of the acetabulum and extended to the iliac crest, the incisura ischiadica major, and the rear acetabulum. The distribution and maximum value of stress were consistent with those reported in a previous study (Zhang et al., 2010). The displacements under the eight loading conditions agreed not only with those in an experimental study but also with those in some computational studies, which are shown in Figure 3 (Miller et al., 1987; Eichenseer et al., 2011; Kim et al., 2014).

The validation results of this model were consistent with those of previous studies, indicating that it was a valid model.

### 3.3 Pelvic and SIJ stress

**FIGURE 4:**
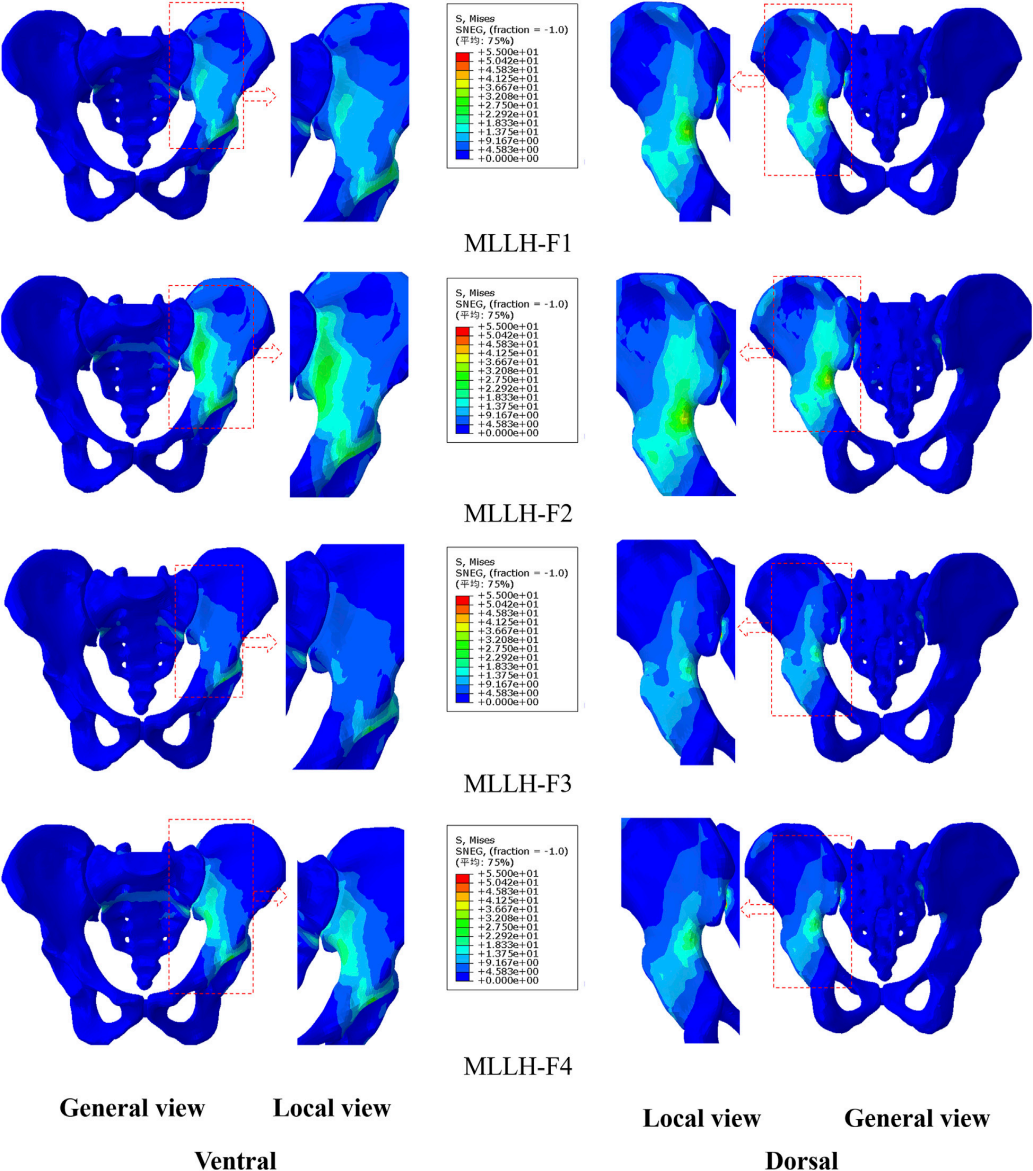
The stress distribution of the pelvis under four MLLHs. The images of the local view are enlarged images in the red box of the general view. MLLH, manipulation of lower limb hyperextension.

The stress distributions of the pelvis under the four MLLHs are shown in Figure 4. In the ventral pelvis, the areas of high stress were located at the left SIJ, arcuate line, and left acetabulum under MLLH-F3 and -F4, while the highly stressed areas extended to the left wing of the ilium under MLLH-F1 and -F2. In the dorsal pelvis, the areas of high stress were located at the left greater ischial notch and left acetabulum under MLLH-F3 and -F4, while these areas extended to the posterior inferior iliac spine under MLLH-F1 and -F2. The maximum stress values produced by MLLH-F1, -F2, -F3 and -F4 were 49.2, 50.5, 48.6 and 54.0 MPa, respectively.

**FIGURE 5:**
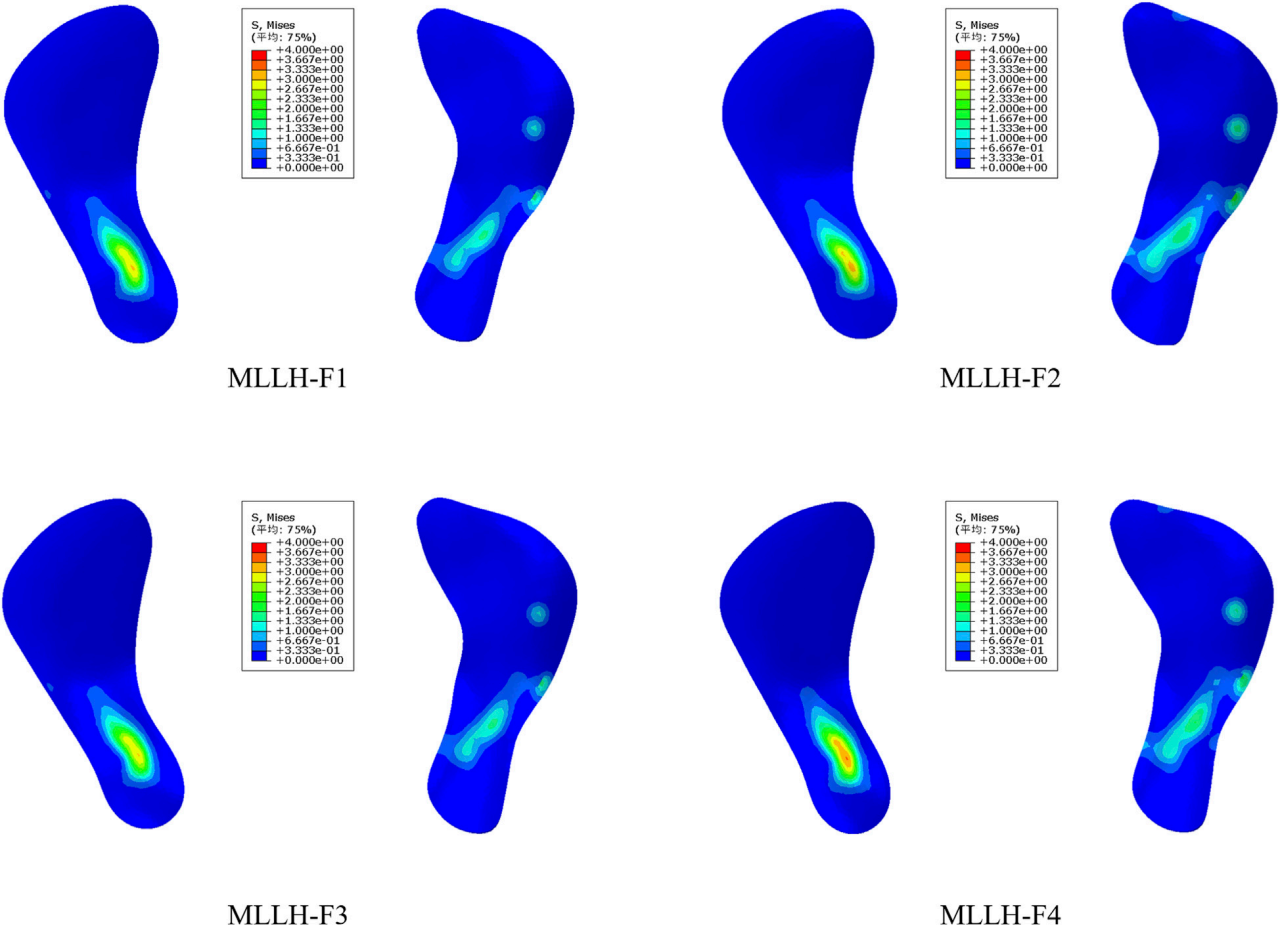
The stress distribution of the SIJ surface of the sacrum under four MLLHs. SIJ, sacroiliac joint; MLLH, manipulation of lower limb hyperextension.

The stress distributions on the sacrum surface of the SIJ are shown in Figure 5. Under all four MLLHs, the areas of high stress were located at the anterior and inferior parts of the SIJ. The stresses on the left SIJ were higher than those on the right SIJ. The maximum stress values produced by MLLH-F1, -F2, -F3 and -F4 were 3.1, 3.2, 3.0 and 3.4 MPa, respectively.

These results indicated that different MLLH techniques had a significant impact on the stress distribution of the pelvis and SIJ, with MLLH-F4 producing the highest stress value.

### 3.4 Displacement of SIJ

**FIGURE 6:**
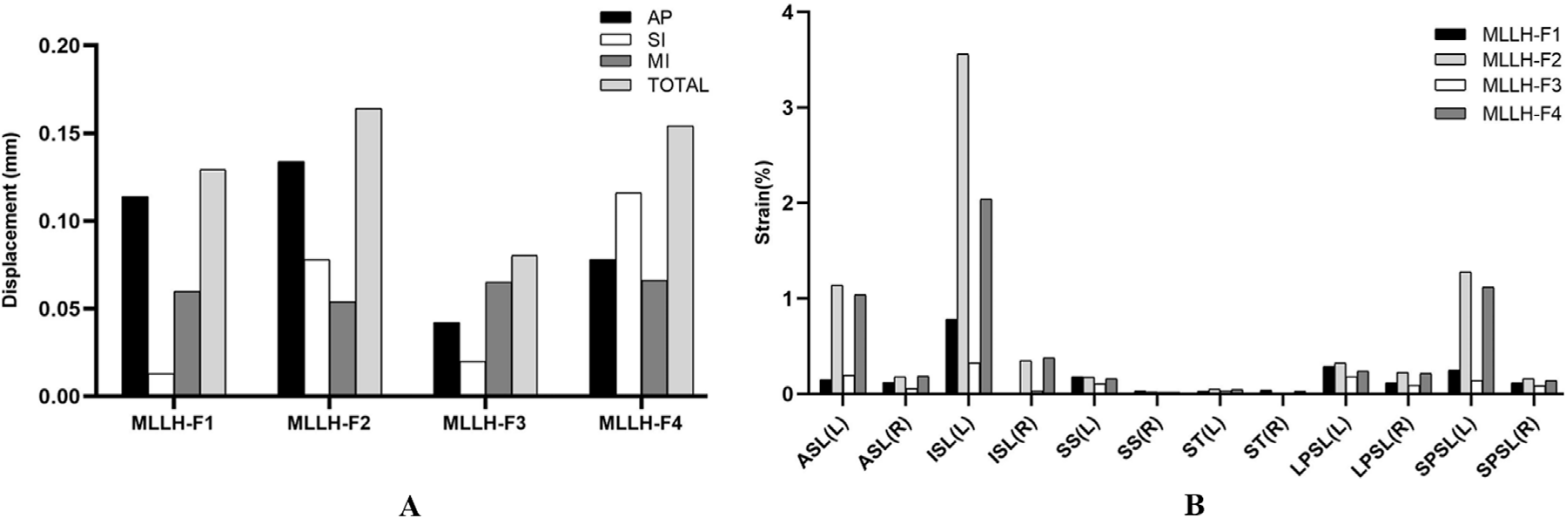
The picture of the left SIJ displacements and the ligament strains under four MLLHs. **(A, B)** Showed the left SIJ displacements and the ligament strains under four MLLHs, respectively. AP, anterior-posterior direction; SI, superior-inferior direction; MI, medial-lateral direction; TOTAL, total displacement; SIJ, sacroiliac joint; MLLH, manipulation of lower limb hyperextension; L, left; R, right; ASL, anterior sacroiliac ligament; ISL, interosseous sacroiliac ligament; SS, sacrospinous ligament; ST, sacrotuberous ligament; LPSL, long posterior sacroiliac ligament; SPSL, short posterior sacroiliac ligament.

Under MLLH-F1, the displacements of the left SIJ were 0.114, 0.013 and 0.060 mm in the anterior-posterior (AP), superior-inferior (SI) and medial-lateral (MI) directions, respectively. In MLLH-F2, the displacements were 0.134, 0.078 and 0.054 mm in the AP, SI and MI directions, respectively. In MLLH-F3, the displacements were 0.042, 0.020 and 0.065 mm in the AP, SI and MI directions, respectively. In MLLH-F4, the displacements were 0.078, 0.116 and 0.066 mm in the AP, SI and MI directions, respectively. The total displacements of the SIJ were 0.129, 0.164, 0.080 and 0.154 mm under MLLH-F1, -F2, -F3 and -F4, respectively. The displacements of the left SIJ are shown in Figure 6A.

These results indicated that different MLLH techniques had a significant impact on the displacement of left SIJ. The MLLH-F2 produced the maximum displacement in AP, as well as the greatest total displacement. The MLLH-F4 produced the maximum displacement in SI and MI.

### 3.5 Strain of ligaments

The ligament strains under the four MLLHs are shown in Figure 6B. In most ligaments, the left ligament strain was greater under each MLLH. With MLLH-F1, the left ISL, LPSL and SPSL were the ligaments with the highest strain values, at 0.78%, 0.29% and 0.25%, respectively. With MLLH-F2, the left ISL, SPSL and ASL had the highest strain values, at 3.56%, 1.28% and 1.14%, respectively. Under MLLH-F3, the left ISL, ASL and LPSL had the highest strain values, at 0.33%, 0.20% and 0.18%, respectively. Under MLLH-F4, the left ISL, SPSL and ASL had the highest strain values, at 2.04%, 1.12% and 1.04%, respectively.

These results indicated that different MLLH techniques had a significant impact on the ligament strains. The MLLH-F2 and -F4 produced the higher ligament strains. The ISL, SPSL, ASL had higher strain values.

## 4 Discussion

SIJ pain is a common disease, affecting 90% of adults throughout their lives (Joukar et al., 2018). SIJ dysfunction is one of the primary etiological factors contributing to SIJ pain. The main causes include:

1. Acute trauma and chronic strain: injuries from accidents, falls, or repetitive stress can lead to joint misalignment or ligament damage.
2. Pregnancy and childbirth: hormonal changes, such as increased relaxin levels, and the physical stress of childbirth can reduce joint stability.
3. Degenerative changes and arthritis: conditions such as osteoarthritis or ankylosing spondylitis can cause inflammation and degenerative changes in the SIJ.
4. Leg length discrepancy and scoliosis.
5. Infectious sacroiliitis: bacterial or viral infections can lead to inflammation of the SIJ.
6. Pelvic or spinal surgery: surgical interventions may alter pelvic mechanics, affecting SIJ function.
7. Other factors: obesity and aging can exacerbate joint degeneration and increase the risk of dysfunction. These factors highlight the multifactorial nature of SIJ dysfunction and its role in the development of SIJ pain. MLLH is a common treatment for SIJ dysfunction without specific causes. However, the best point and direction of the manipulative force are currently disputed. Thus, this work intended to study the four MLLHs based on the finite element model to provide a theoretical basis for the manipulation.

Since the force point of MLLH was on the dorsal side of the pelvis, the maximum pelvic stresses produced by the four MLLHs were all on the dorsal side of the pelvis. The area of high stress was mainly located at the greater ischial notch, which was related to the structure of the pelvis. The greater ischial notch is the transition area of the broad iliac wing to the narrow ischial spine and acetabulum. The area of high stresses caused by MLLH-F1 and -F2 extended to the iliac crest, which was related to the fact that the force points of MLLH-F1 and -F2 were on the iliac crest. The maximum pelvic stresses caused by MLLH-F1 and -F3 were smaller than those caused by MLLH-F2 and -F4. This difference was related to the direction of manipulative force, which was roughly parallel to the SIJ surface in the former.

It is difficult to measure the stress on the surface of the SIJ in the human body. Shi et al. (2014) built a pelvic model and found that the stresses of the SIJ surface of the sacrum and ilium were 16.5 and 31.4 MPa under the double-support standing posture. Zhang et al. (2022) observed that the stresses of the right SIJ surface of the sacrum ranged from 15 to 21 MPa, 18–19 MPa and 17–20 MPa under flexion and extension, lateral bending and rotation motions, respectively. Kiapour et al. (2012) studied the relationship between the load distribution on the SIJ surface and the limb length discrepancy. For flexion and extension, lateral bending and rotation motions, the respective stresses of the SIJ surface ranged from 3.6 to 6.9 MPa, 4.5–53.1 MPa and 24.7–83.5 MPa under various leg length discrepancies (0, 1, and 2 cm). In this study, the stress on the left SIJ surface was greater than that on the right, which was related to the manipulative force on the left ilium. MLLH-F4 produced the highest stress, at 3.4 MPa, while MLLH-F3 produced the lowest stress, at 3.0 MPa. The stresses on the SIJ were less than those of normal activity of humans, so the MLLH would not cause damage to the SIJ. The SIJ surface of the ilium was behind, outside and below the SIJ surface of the sacrum. MLLH-F4 made the ilium nearer to the sacrum. MLLH-F3 caused the ilium to move nearer to the sacrum parallel to it. Therefore, the stress produced by MLLH-F4 was higher than that produced by MLLH-F3. The anterior and inferior parts of the SIJ surface were the synovial structure, and the posterior part was the ligament structure, so the stress on the SIJ was concentrated on the anterior and inferior parts. This result was consistent with that of Kim’s study (Kim et al., 2014).

Walker (1992) found that SIJ displacements were no more than 3 mm and rotation was less than 2° when SIJ underwent rotation or flexion-extension in a standing and sitting position. Klima et al. (2018) performed a cadaveric study and found that the SIJ displacements were 0.1, 0.0, and 0.3 mm in the AP, MI, and SI directions under 100% body weight loading. Jacob and Kissling (1995) performed an *in vivo* study using a cam k-wire device. The results showed that SIJ displacements were 0.4, 0.7, and 0.5 mm in the AP, MI, and SI directions when the SIJ underwent flexion-extension in a one-legged stance in an upright position. Kibsgård et al. (2012) observed that the SIJ displacements were 0.5, 0.4, and 0.3 mm in the AP, MI, and SI directions using Roentgen stereophotogrammetry analysis when the SIJ underwent flexion-extension, lateral bending and rotation. In this study, it was found that the SIJ displacements were less than 0.2 mm under all four MLLHs, which was consistent with previous studies (Walker, 1992; Jacob and Kissling, 1995; Kibsgård et al., 2012; Klima et al., 2018). These small displacements are within the normal range of motion and are not harmful to the SIJ. The SIJ is an incomplete sagittal and coronal joint, so its motion is complex. Under MLLH-F1 and -F2, the displacement in the AP direction was the largest. Under MLLH-F3, the displacement in the MI direction was the largest. Under MLLH-F4, the displacement in the SI direction was the largest. Thus, the point and direction of the manipulative force had clear influences on the SIJ movement. In addition, MLLH-F2 produced the largest total displacement among the four MLLHs. This might be related to the greater torque of SIJ rotation caused by MLLH-F2.

Hammer et al. (2013) built a finite element model and indicated that an increase in SIJ cartilage and ligament material stiffnesses decreased pelvic motion. Enix and Mayer (2019) suggested that hypermobility of the SIJ could be caused by ligamentous instability or be secondary to adaptive biomechanical changes and increased stresses affecting the joints of the pelvis. Hammer et al. (2019) found that the SS and ST played an important role in maintaining SIJ stability in the two-leg stance. The instability resulting from partial or complete SS and ST injury merits consideration when choosing and designing treatment strategies. Ligaments play an important role in maintaining pelvic stability. Our results indicated that the strains of the ISL, SPSL, and ASL were larger than those of the other three ligaments under all four MLLHs. The ISL and SPSL are located at the posterior and upper parts of the SIJ. When the MLLHs were performed, the ISL and SPSL were the first to withstand the force. The force point of the MLLH-F2 was closer to the synovial portion of the SIJ, resulting in a shorter lever arm, which made it easier to produce ligament strain. Additionally, the direction of the manipulative force was parallel to the sagittal plane of body and intersected with the orientation of the ISL, SPSL and ASL, further facilitating the induction of ligament strain. This biomechanical advantage may enhance the effectiveness of the manipulation in restoring joint alignment and improving ligament function.

Low back pain caused by SIJ dysfunction primarily attributes to minor joint subluxation and abnormal strain in the surrounding ligaments. Manipulation aims to alleviate pain and restore function by realigning the joint surfaces and normalizing the mechanical state of the ligaments, which forms the biomechanical basis for its effectiveness. This study found that among four MLLHs, MLLH-F2 resulted in the greatest displacement of the SIJ and induced the maximum strain in the ligaments. These findings suggested that MLLH-F2 was highly effective in restoring joint alignment and improving the mechanical state of the ligaments. In clinical, when applying the MLLH, the midpoint between the highest point of the iliac crest and the PSIS should be selected as the manipulative force point. The direction of manipulative force should be parallel to the sagittal plane of the body. This approach can optimize the therapeutic effect of MLLH by ensuring precise force transmission to the SIJ, and improve clinical outcomes in the treatment of SIJ dysfunction.

There are some limitations to this experiment. First, the data used in this model were derived from a young male individual, which may not be applicable to female or elderly populations. Therefore, the findings of this study may have limited generalizability to these groups. Future research should focus on investigating the characteristics of other populations. Second, in this model, ligaments were typically simplified as linear elements, despite the fact that ligaments exhibited nonlinear characteristics in reality. This simplification might result in the model’s inability to accurately reflect the true mechanical behavior of ligaments. Furthermore, the model did not account for muscle factors, which represented a significant deviation from actual conditions. The manipulation in the model was relatively simplistic, whereas clinical manipulative procedures were far more complex, involving greater detail and dynamic adjustments. Therefore, although the model held certain values in research and prediction, the findings must be interpreted with caution and validated against clinical practice. Future improvements to the model could consider incorporating nonlinear ligament models and muscle factors to more accurately simulate real-world conditions. Finally, the SIJs in this model were normal joint structures, but MLLH was applied to subluxated SIJs clinically. The results might not fully reflect the mechanical characteristics of MLLH.

## 5 Conclusion

This study analyzed the effects of four MLLHs on the SIJ. MLLH-F2 and -F4 caused greater stresses on the pelvis and the SIJ surface. The high stress areas of the SIJ were located at the anterior and inferior parts of the SIJ. The four MLLHs all produced small SIJ displacements. Among them, MLLH-F2 and -F4 produced greater total displacements. MLLH-F1 and -F2 mainly produced displacement in the AP direction. MLLH-F3 mainly produced displacement in the MI direction. MLLH-F4 mainly produced displacement in the SI direction. In addition, the four MLLHs all caused different degrees of ligament strain, MLLH-F2 the greatest.

## Data Availability

The original contributions presented in the study are included in the article/supplementary material, further inquiries can be directed to the corresponding authors.

## Abbreviations

MLLHs, Manipulations of lower limb hyperextension; SIJ, sacroiliac joint; ASL, anterior sacroiliac ligament; SPSL, short posterior sacroiliac ligament; LPSL, long posterior sacroiliac ligament; SS, sacrospinous ligament; ISL, interosseous sacroiliac ligament; ST, sacrotuberous ligament; PSIS, posterior superior iliac spine; AP, anterior-posterior; SI, superior-inferior; MI: medial-lateral.

## References

[B1] Al-Subahi M. Alayat M. Alshehri M. A. Helal O. Alhasan H. Alalawi A. (2017). The effectiveness of physiotherapy interventions for sacroiliac joint dysfunction: a systematic review. J. Phys. Ther. Sci. 29, 1689–1694. 10.1589/jpts.29.1689 28932014 PMC5599847

[B2] Cohen S. P. (2005). Sacroiliac joint pain: a comprehensive review of anatomy, diagnosis, and treatment. Anesth. Analgesia 101, 1440–1453. 10.1213/01.ane.0000180831.60169.ea 16244008

[B3] Dontigny R. L. (1990). Anterior dysfunction of the sacroiliac joint as a major factor in the etiology of idiopathic low back pain syndrome. Phys. Ther. 70, 250–262. 10.1093/ptj/70.4.250 2138334

[B4] Eichenseer P. H. Sybert D. R. Cotton J. R. (2011). A finite element analysis of sacroiliac joint ligaments in response to different loading conditions. Spine 36, E1446–E1452. 10.1097/brs.0b013e31820bc705 21311405

[B5] Enix D. E. Mayer J. M. (2019). Sacroiliac joint hypermobility biomechanics and what it means for health care providers and patients. PM and R J. Inj. Funct. Rehabilitation 11 (Suppl. 1), S32–S39. 10.1002/pmrj.12176 31025539

[B6] Fan Z. Wu S. (2021). Based on the theory of ‘bone staggered suture and sinew grooving’, the characteristics of Lin's bone-setting manipulation in the treatment of sacroiliac joint dysfunction. J. Trad. Chin. Orthop. Trauma 33, 47–49.

[B7] Farazdaghi M. R. Motealleh A. Abtahi F. Panjan A. Šarabon N. Ghaffarinejad F. (2018). Effect of sacroiliac manipulation on postural sway in quiet standing: a randomized controlled trial. Braz. J. Phys. Ther. 22, 120–126. 10.1016/j.bjpt.2017.09.002 28993042 PMC5883953

[B8] García-Peñalver U. J. Palop-Montoro M. V. Manzano-Sánchez D. (2020). Effectiveness of the muscle energy technique versus osteopathic manipulation in the treatment of sacroiliac joint dysfunction in athletes. Int. J. Environ. Res. Public Health 17, 4490. 10.3390/ijerph17124490 32580480 PMC7345493

[B9] Hammer N. Höch A. Klima S. Le Joncour J.-B. Rouquette C. Ramezani M. (2019). Effects of cutting the sacrospinous and sacrotuberous ligaments. Clin. Anat. (New York, N.Y.) 32, 231–237. 10.1002/ca.23291 30281852

[B10] Hammer N. Steinke H. Lingslebe U. Bechmann I. Josten C. Slowik V. (2013). Ligamentous influence in pelvic load distribution. Spine J. Official J. North Am. Spine Soc. 13, 1321–1330. 10.1016/j.spinee.2013.03.050 23755919

[B11] Hing W. Hall T. Rivett D. Vicenzino B. (2015). The mulligan concept of manual therapy: textbook of techniques. Elsevier Health Sciences.

[B12] Huang J. He Y. Liu H. He Z. Wu B. Zhao X. (2019). Advances in the diagnosis, treatment and research of sacroiliac joint misalignment. Tissue Eng. Res. China 23, 3201–3206.

[B13] Jacob H. a.C. Kissling R. O. (1995). The mobility of the sacroiliac joints in healthy volunteers between 20 and 50 years of age. Clin. Biomech. (Bristol, Avon) 10, 352–361. 10.1016/0268-0033(95)00003-4 11415579

[B14] Joukar A. Shah A. Kiapour A. Vosoughi A. S. Duhon B. Agarwal A. K. (2018). Sex specific sacroiliac joint biomechanics during standing upright: a finite element study. Spine 43, E1053–E1060. 10.1097/brs.0000000000002623 29509655

[B15] Kamali F. Zamanlou M. Ghanbari A. Alipour A. Bervis S. (2019). Comparison of manipulation and stabilization exercises in patients with sacroiliac joint dysfunction patients: a randomized clinical trial. J. Bodyw. Mov. Ther. 23, 177–182. 10.1016/j.jbmt.2018.01.014 30691749

[B16] Kiapour A. Abdelgawad A. A. Goel V. K. Souccar A. Terai T. Ebraheim N. A. (2012). Relationship between limb length discrepancy and load distribution across the sacroiliac joint-a finite element study. J. Orthop. Res. 30, 1577–1580. 10.1002/jor.22119 22488899

[B17] Kibsgård T. J. Røise O. Stuge B. Röhrl S. M. (2012). Precision and accuracy measurement of radiostereometric analysis applied to movement of the sacroiliac joint. Clin. Orthop. Relat. Res. 470, 3187–3194. 10.1007/s11999-012-2413-5 22695864 PMC3462864

[B18] Kim Y. H. Yao Z. Kim K. Park W. M. (2014). Quantitative investigation of ligament strains during physical tests for sacroiliac joint pain using finite element analysis. Man. Ther. 19, 235–241. 10.1016/j.math.2013.11.003 24378472

[B19] Klima S. Grunert R. Ondruschka B. Scholze M. Seidel T. Werner M. (2018). Pelvic orthosis effects on posterior pelvis kinematics an *in-vitro* biomechanical study. Sci. Rep. 8, 15980. 10.1038/s41598-018-34387-7 30374032 PMC6206162

[B20] Lee C.-H. Hsu C.-C. Huang P.-Y. (2017). Biomechanical study of different fixation techniques for the treatment of sacroiliac joint injuries using finite element analyses and biomechanical tests. Comput. Biol. Med. 87, 250–257. 10.1016/j.compbiomed.2017.06.007 28618337

[B21] Lindsey D. P. Perez-Orribo L. Rodriguez-Martinez N. Reyes P. M. Newcomb A. Cable A. (2014). Evaluation of a minimally invasive procedure for sacroiliac joint fusion – an *in vitro* biomechanical analysis of initial and cycled properties. Med. Devices Auckl. N.Z. 7, 131–137. 10.2147/mder.s63499 PMC4031207 24868175

[B22] Liu G. Feng W. Mak F.-W. (2020). Clinical observation of Lin's manipulation combined with musculoskeletal ultrasound in the treatment of postpartum sacro-iliac articulation malposition. J. Pract. traditional Chin. Med. 36, 1065–1067.

[B23] Mclauchlan G. J. Gardner D. L. (2002). Sacral and iliac articular cartilage thickness and cellularity: relationship to subchondral bone end-plate thickness and cancellous bone density. Rheumatol. Oxf. Engl. 41, 375–380. 10.1093/rheumatology/41.4.375 11961166

[B24] Miller J. A. Schultz A. B. Andersson G. B. (1987). Load-displacement behavior of sacroiliac joints. J. Orthop. Res. 5, 92–101. 10.1002/jor.1100050112 3819915

[B25] Nejati P. Safarcherati A. Karimi F. (2019). Effectiveness of exercise therapy and manipulation on sacroiliac joint dysfunction: a randomized controlled trial. Pain Physician 22, 53–61. 30700068

[B26] Poilliot A. J. Zwirner J. Doyle T. Hammer N. (2019). A systematic review of the normal sacroiliac joint anatomy and adjacent tissues for pain physicians. Pain Physician 22, E247–E274. 31337164

[B27] Schuit D. Mcpoil T. G. Mulesa P. (1989). Incidence of sacroiliac joint malalignment in leg length discrepancies. J. Am. Podiatric Med. Assoc. 79, 380–383. 10.7547/87507315-79-8-380 2810074

[B28] Shi D. Wang F. Wang D. Li X. Wang Q. (2014). 3-D finite element analysis of the influence of synovial condition in sacroiliac joint on the load transmission in human pelvic system. Med. Eng. and Phys. 36, 745–753. 10.1016/j.medengphy.2014.01.002 24508529

[B29] Vanelderen P. Szadek K. Cohen S. P. De Witte J. Lataster A. Patijn J. (2010). 13. Sacroiliac joint pain. Pain Pract. Official J. World Inst. Pain 10, 470–478. 10.1111/j.1533-2500.2010.00394.x 20667026

[B30] Walker J. M. (1992). The sacroiliac joint: a critical review. Phys. Ther. 72, 903–916. 10.1093/ptj/72.12.903 1454866

[B31] Xiao P. Li Y. Yan Y. Hou D. Zhang K. Xu Z. (2022). CT imaging anatomical observations of the sacroiliac joint space in normal people of different ages. Chin. J. Clin. Anat. 40, 143–149. 10.13418/j.issn.1001-165x.2022.2.05

[B32] Xie H. (2017). Clinical study on sacroiliac joint dysfuntion treated by manipulation. world Clin. Med. 11, 140–142.

[B33] Xu Z. Li Y. Zhang S. Liao L. Wu K. Feng Z. (2020). A finite element analysis of sacroiliac joint displacements and ligament strains in response to three manipulations. BMC Musculoskelet. Disord. 21, 709. 10.1186/s12891-020-03735-y 33115467 PMC7594473

[B34] Zhang Q.-H. Wang J.-Y. Lupton C. Heaton-Adegbile P. Guo Z.-X. Liu Q. (2010). A subject-specific pelvic bone model and its application to cemented acetabular replacements. J. Biomechanics 43, 2722–2727. 10.1016/j.jbiomech.2010.06.023 20655051

[B35] Zhang S. Chen Y. Ren R. Jiang S. Cao Y. Li Y. (2022). Quantitative study on the biomechanical mechanism of sacroiliac joint subluxation: a finite element study. J. Orthop. Res. Official Publ. Orthop. Res. Soc. 40, 1223–1235. 10.1002/jor.25132 34185334

[B36] Zhang S. Feng Z. Chen Y. Zhang L. Qi J. Li Y. (2019). CT imaging anatomical observations of the normal sacroiliac joint space in adults and its clinical significance. Chin. J. Clin. Anat. 37, 14–19. 10.13418/j.issn.1001-165x.2019.01.004

[B37] Zhong Z. Liao L. Li Z. Xu X. Miao X. Zhang K. (2025). Clinical study on the treatment of sacroiliac joint subluxation by tuina manipulation combined with pelvic floor biofeedback. Mod. Chin. Med. Clin., 1–12.

